# Electromagnetic fields modulate neuronal membrane ionic currents through altered cellular calcium homeostasis

**DOI:** 10.1111/nyas.15386

**Published:** 2025-06-26

**Authors:** Federico Bertagna, Shiraz Ahmad, Rebecca Lewis, S. Ravi P. Silva, Johnjoe McFadden, Christopher L.‐H. Huang, Hugh R. Matthews, Kamalan Jeevaratnam

**Affiliations:** ^1^ Leverhulme Quantum Biology Doctoral Training Centre University of Surrey Guildford UK; ^2^ School of Veterinary Medicine, Faculty of Health and Medical Sciences University of Surrey Guildford UK; ^3^ Advanced Technology Institute University of Surrey Guildford UK; ^4^ School of Biosciences and Medicine, Faculty of Health and Medical Sciences University of Surrey Guildford UK; ^5^ Physiological Laboratory University of Cambridge Cambridge UK

**Keywords:** calcium homeostasis, electromagnetic fields, hippocampus, ion channels, patch clamp

## Abstract

The biological effects of electromagnetic fields (EMFs) on the central nervous system (CNS) have been widely reported in the literature. Their nature and extent are thought to depend on parameters such as field intensity and frequency. Of these, extremely low‐frequency (50 Hz) fields have been reported to influence neuronal firing in CNS regions, including the hippocampus. We applied the loose patch clamp technique to study the effects of 1 mT exposures of such fields over the course of 60 min on cornus ammonis 1 (CA1) pyramidal neuron membranes in coronal hippocampal slices. Such exposure decreased both inward and transient outward currents. Pharmacological blockers of ryanodine receptor (RyR)‐dependent Ca^2+^ release (dantrolene) and endoplasmic reticular Ca^2+^ store reuptake (SERCA; cyclopiazonic acid) both abrogated these effects. We thus implicate Ca^2+^ homeostasis in an EMF‐induced modulation of neuronal excitability through its regulation of voltage‐gated channels.

## Graphical abstract

Electromagnetic fields (50 Hz, 1 mT) affect hippocampal CA1 neuron excitability by reducing membrane currents. These effects, blocked by agents targeting calcium signaling pathways, suggest EMFs influence neuronal function via disruption of Ca^2^⁺ homeostasis and voltage‐gated channel regulation.

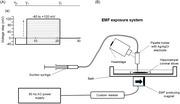

## INTRODUCTION

Electromagnetic fields (EMFs) have been reported to exert effects on plant and animal, including human, development and physiology,^1^, ^2^, ^3^, ^4^ and demonstrate therapeutic potential in wound healing^5^, ^6^ and diseases, including cancer.^7^, ^8^ In the central nervous system (CNS), transcranial magnetic stimulation^9^ has been used in the clinical management of neurological conditions, including ischemic stroke^10^ and Parkinson's disease.^11^ Experimental efforts to elucidate the mechanisms underlying their CNS effects have mostly utilized in vitro model systems, including brain slices.^12^, ^13^ Several reports^14^, ^15^, ^16^, ^17^ have implicated altered intracellular Ca^2+^ homeostasis in these effects. The latter varied with field intensity, duration, and frequency.^1^ Recent attention has been directed at extremely low‐frequency (<300 Hz)^18^ magnetic fields. These are of low energy and, therefore, do not generate thermal effects, but their induced currents could potentially modify tissue homeostasis.^19^

Among different EMF frequencies tested, 50 Hz has been frequently explored in the biological literature.^1^ This represents the most frequent source of exposure from power lines and domestic installations.^20^ Fifty Hertz magnetic fields were recently reported to alter both synaptic plasticity and the development of long‐term potentiation in both adult^21^ and new‐born^22^ rats, and enhance neurogenesis in the mouse hippocampus.^23^ These effects have been attributed to acute, direct^24^ or indirect,^14^, ^17^, ^25^ actions on ion channels, including voltage‐gated Na^+^ channels (Na_v_),^26^ high threshold voltage‐gated Ca^2+^ channels (Ca_v_), and Ca^2+^‐activated potassium channels (K_Ca_).^27^ Both acute and chronic exposure to 50 Hz magnetic fields were reported to increase Ca^2+^ influx across the surface membrane of various hippocampal neuronal types,^15^, ^27^, ^28^ altering Ca^2+^ signaling and synaptic plasticity.^29^ Other recent reports alternatively implicated mobilization of internal mitochondrial and endoplasmic reticular (ER) Ca^2+^ stores (ICS)^30^ in the observed increases in [Ca^2+^]_i_.^25^ Ca^2+^‐signaling is pivotal in neuronal physiology, and regulates other key processes, including neural differentiation, survival, and apoptosis.^31^ Previous findings showed that 50 Hz magnetic fields increased [Ca^2+^]_i_ in HEK 293 cells. These effects were diminished when ER Ca^2+^‐dependent ATPase (SERCA)‐mediated transport activity was blocked.^17^ However, other studies reported no increase in [Ca^2+^]_i_ after either acute or chronic exposure to low‐frequency EMFs.^32^, ^33^

Here, we use the loose patch clamp technique to study the effects of low‐frequency (50 Hz) EMF on membrane currents elicited by standard depolarization protocols^34^ in murine CA1 pyramidal neurons. We specifically examined the recently reported functional Na^+^ channel modification by alterations in intracellular Ca^2+^ homeostasis. The latter includes diffusive and reaction processes known to change with significantly longer time courses, as observed here, than the more immediate excitable Na^+^ channel changes. The possible importance of this feedback effect is reflected in its occurrence not only in the hippocampal neurons^35^ studied here, but also in other excitable, skeletal,^36^ and cardiac^37^ muscle.^38^, ^39^ In all these, the ryanodine receptor (RyR) and endoplasmic reticular Ca^2+^ transport (SERCA) play a major role. These are specifically targetable, respectively, by the pharmacological agents dantrolene^40^ and cyclopiazonic acid (CPA).^41^ Such antagonism provided an opportunity to correlate the observed changes with alterations in Ca^2+^ homeostasis in intact native neurons. We thereby implicated Ca^2+^ homeostasis in an EMF‐induced modulation of neuronal excitability (see Ref. 17) through the regulation of voltage‐gated channels.

## MATERIALS AND METHODS

All experimental procedures were approved by and conformed with the guidelines of the animal experiments ethical committee of the University of Surrey, Guildford, UK (NASPA‐1819‐25). All chemical compounds used were purchased from Sigma‐Aldrich unless otherwise stated.

### Animals

Four‐week‐old C57BL/6 male mice (Charles River UK Ltd.) were kept under monitored conditions (ambient temperature 23 ± 2°C, 12‐h light/dark cycle) and alimented through food pellets and water supplied *ad libitum*. Experimental groups were subject to at least 1 week of accommodation in animal house conditions prior to experiments. On the day of the experiment, a single animal was sacrificed by cervical dislocation [Schedule I, UK Animals (Scientific Procedures) Act 1986] and the brain was immediately extracted and sectioned.

### Tissue preparation

Tissue was processed as previously described.^34^ Briefly, the brain was immediately removed after cervical dislocation and immersed in ice‐cold HEPES holding artificial cerebrospinal fluid (aCSF)^42^ containing (mM) 92 NaCl, 2.5 KCl, 30 NaHCO_3_, 1.25 NaH_2_PO_4_, 20 HEPES, 25 glucose, 10 MgCl_2_, and 0.5 CaCl_2_, with pH adjusted to 7.4, constantly bubbled with a mixture of 95% O_2_–5% CO_2_,^42^ and transported to the slicing station. Here, 300‐µm‐thick coronal hippocampal slices were obtained using a micro‐slicer (7000smz‐2 Vibratome, Campden Instruments Ltd.). Samples were equilibrated for 1 h at room temperature (20–25°C) in HEPES holding aCSF, constantly bubbled with 95% O_2_–5% CO_2_.

From a single brain sample, four coronal slices were obtained corresponding to the anterior and medial hippocampal region. From each slice, a single patch was obtained and studied. The slices not immediately used for the experiment were kept in HEPES holding aCSF, constantly bubbled with a mixture of 95% O_2_–5% CO_2_ for up to 5 h.

### Bath setup and perfusion apparatus

The specifics of bath setup and perfusion apparatus are discussed elsewhere.^34^ Briefly, a single slice was located and immersed in 30 mL of standard recording aCSF containing (mM): 124 NaCl, 2.5 KCl, 1.25 NaH_2_PO_4_, 24 NaHCO_3_, 5 HEPES, 12.5 glucose, 2 MgCl_2_, 2 CaCl_2_, with pH 7.3−7.4 and T = 23–25°C. Solution entered and left the bath via two peristaltic pumps (model 101UR, Watson‐Marlow), both regulated at a 4 mL/min flow rate to minimize disturbance on the tissue. Prior to perfusion, solutions were equilibrated for at least 1 h at room temperature and constantly bubbled with a mixture of 95% O_2_–5% CO_2_.

### Loose patch pipette manufacture and deployment

The technicalities of pipette fabrication and deployment are discussed in Ref. 34. Briefly, patch pipettes were manufactured from borosilicate glass capillary tubes (GC150‐10; Harvard Apparatus) through a horizontal micropipette puller (Model P‐97 Sutter Instrument Co.), achieving progressive taper. Only electrodes with a square‐ended tip with a 20–25 µm diameter were selected. The pipette was inserted into a 45° inclined pipette holder (model Q45W‐B15P, Warner Instruments), held at 45° to achieve perpendicular contact with the surface of the slice. In standard recording aCSF, the mean pipette resistance (R_pip_) recorded was ∼ 200 kΩ. The pipette tip was dipped into the bath solution, drawing it up into the pipette until it made contact with the enclosed Ag/AgCl wire, while still maintaining an air gap within the electrode shaft. The offset potential of the bath's active ground was then tuned until the pipette electrode registered zero current.

### Loose patch clamp recording

The details of loose patch clamp recording employing a custom‐built loose patch amplifier circuit are discussed in Ref. 34. This separated membrane from leak currents and additionally balanced out voltage drops arising from pipette currents, prior to feeding the resulting signal into the voltage clamp circuitry. These involved adjusting variable resistances in a compensating bridge circuit to match the voltage drops across the pipette (*R*_pip_) and seal resistance (*R*_seal_). These ensured that the membrane patch was clamped to the command potential, and the circuit output corresponded to the membrane current flowing through the patch only. The command voltage clamp steps were applied using an IBM‐compatible computer. The loose patch technique applies its voltage clamp steps to the pipette solution at the extracellular rather than the intracellular face of the membrane patch. These applied voltage steps are, therefore, of opposite sign to the conventionally expressed membrane potential. The resulting membrane potentials are, therefore, reported relative to the resting membrane potential (RMP). A series of membrane‐depolarizing clamp steps was used to produce membrane depolarization and derive current–voltage curves reflecting channel activation.

When the pipette makes contact with the membrane, it induces a shift in the magnitude of the uncompensated currents triggered by low‐amplitude voltage clamp pulses, indicating a rise in resistance at the pipette tip. Negative pressure was then applied to the electrode to stabilize the seal, and the R_seal_ was adjusted accordingly. The presence of membrane currents was explored through a 25 ms voltage step of (RMP + 80) mV. Only patches containing clear‐cut currents were selected. Average R_seal_ varied from patch to patch and ranged between 1.5 and 2.0 times the value of R_pip_ (average ∼ 300 kΩ). Currents were recorded from the stratum pyramidalis of the murine CA1 hippocampus. Here, patches were clamped to a series of increasingly depolarized voltages relative to the RMP. The depolarization‐activated currents produced were studied in physiological conditions, following drug administration, and after different times of exposure (15, 30, 45, 60 min) to EMF.

### EMF production

**FIGURE 1:**
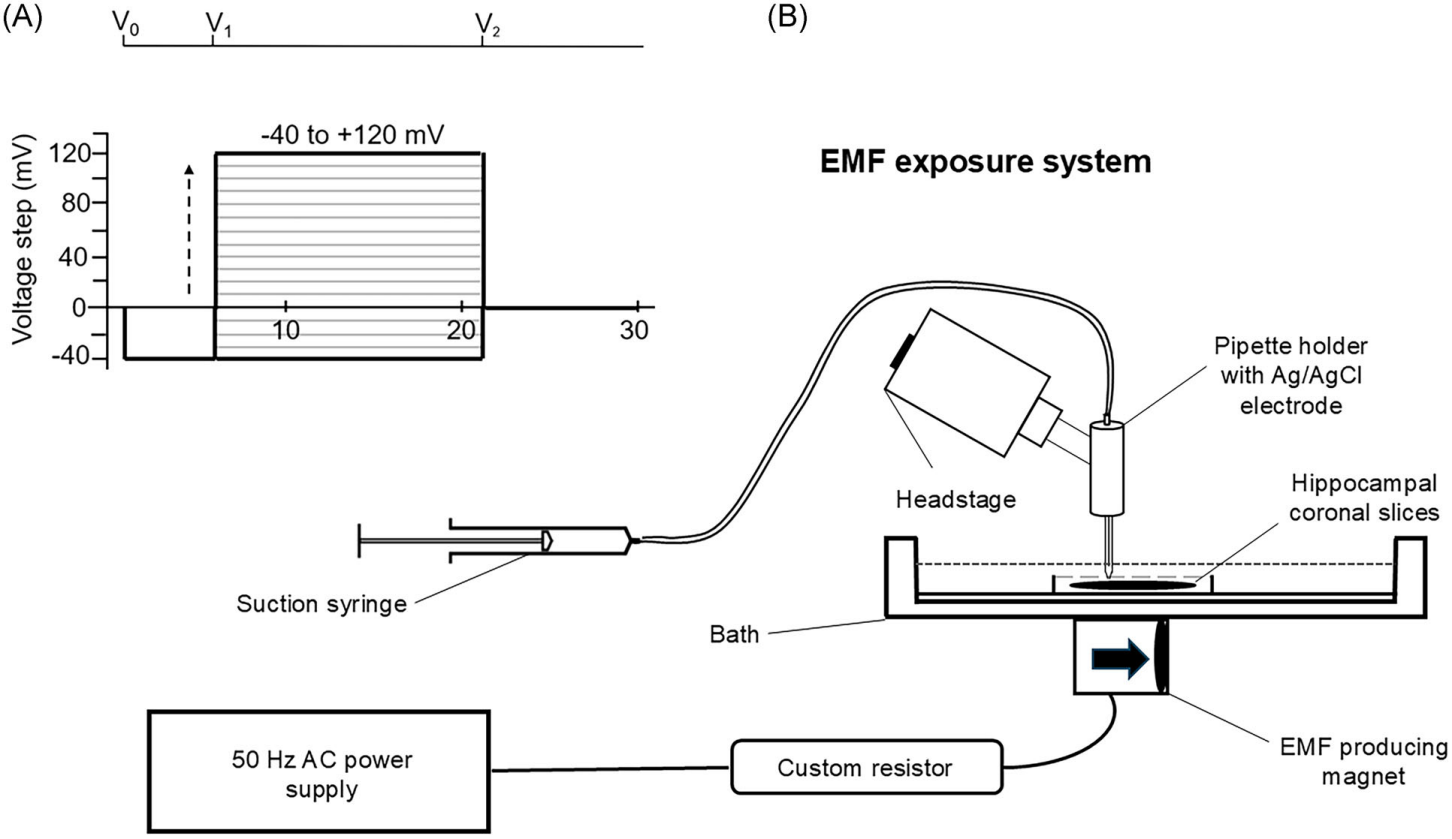
Pulse protocol used in the study and schematic diagram of the experimental setup. (A) Pulse protocol used for voltage dependence of current activation, imposing two voltage steps over a time‐course of 30 ms before restoring resting membrane potential (RMP). A 5 ms hyperpolarizing pre‐pulse (to voltage V_0_) at (RMP – 40) mV relieved any residual Na^+^ channel (Na_v_) inactivation at the RMP. This was followed by a variable test pulse (to voltage V_1_) of 15 ms duration starting at (RMP – 40) mV and altered in (RMP + 10) mV increments until a maximum test voltage of (RMP + 120) mV was reached. The RMP was finally restored after 21 ms of recording time (to voltage V_2_). (B) Front view of the experimental setup showing the amplifier head stage connected to a 45° angled electrode holder, mounted at 45° to the preparation to permit perpendicular contact of the pipette tip with the tissue surface, and the pipette connected to a suction syringe. Electromagnetic fields (EMFs) are produced using a magnet powered by a 50 Hz AC power supply. A custom resistor is used to limit the intensity of the EMF to 1 mT.

The system used to expose hippocampal coronal slices to an EMF (Figure 1B) involved a magnet composed of a solenoid coiled around a 3 mm diameter ferrite core fixed into a nonmetallic mount. This was powered through a 50 Hz AC power supply (Adapter Technology Co., Ltd) that produced the input voltage of the pulse, and the magnetic flux densities could be regulated to 1.0 mT, measured and monitored through a gaussmeter (Hirst Magnetic Instruments Ltd). The intensity was limited to a maximal value of 1 mT through a custom resistor. The magnet was oriented such that the resulting lines of magnetic flux ran parallel to the fluid surface in the recording chamber. The temperature of the extracellular solution, continuously monitored through a thermocouple probe, remained constant over the duration of the experiments, and was identical between the sham and test experiments.

### Experimental protocols

Successive recording runs were separated by time intervals sufficient to permit the patched membranes to regain their previous steady state and, therefore, background RMPs. The patched membrane represents a membrane surface area, defined by the pipette tip diameter. The latter is extremely small relative to the remaining cell surface area to which it is coupled by the electrically conducting extracellular and intracellular fluids. Its RMP would, therefore, tend to that of such a surrounding membrane. While the patch pipette was within the bath prior to each recording run, its internal potential was zeroed by monitoring the patch pipette current to give a holding potential that was equal to the 0 mV bath potential. The initial pipette application to the cell membrane would then leave the patch at the fiber RMP. Following seal formation,  applied pipette currents used in the voltage clamp protocols would only clamp voltages across membrane within the patch.

Each protocol then began with the application of a 5 ms hyperpolarizing step at (RMP − 40) mV imposed at 1 ms to relieve any residual Na_v_ inactivation at the RMP. This was followed by a test pulse of variable amplitude and 15 ms duration, starting at (RMP − 40) mV and altered in +10 mV increments until a maximum test voltage of (RMP + 120) mV was reached (Figure 1A). The RMP was finally restored after 21 ms of recording time (Figure 1A). To correct for any remaining uncompensated leak current, a P/4 procedure was implemented. Here, four voltage clamp steps with opposite polarity, each a quarter of the magnitude of the test pulse, immediately followed the test pulse. Since the P/4 pulses spanned voltage ranges that would not activate any voltage‐gated conductance, they exclusively represented residual uncompensated leak currents but of opposite sign. These leak currents were nullified by summing them with the recorded test pulse current. A sequence of membrane‐depolarizing clamp steps was employed to generate current–voltage curves that depicted channel activation alongside the P/4 pulse procedure.

Data were sampled at a 50 kHz digital sampling rate and filtered with a DC‐10 kHz bandwidth, using a 10 kHz Bessel low‐pass filter. The area of interest was optically viewed using a dissection microscope (Zeiss). All the experiments were carried out at room temperature (20–25°C). The data were digitized and stored using custom‐made loose patch clamp software.

### Drug administration

Once the seal was acquired and stabilized, standard recording aCSF was circulated through the bath chamber containing the specimen by activating the perfusion pumps while monitoring R_seal_. Once a set of control currents was obtained, experimental solutions containing drugs at pre‐established concentrations were perfused. The perfused solution exceeded four times in volume (120 mL) that of the bath (30 mL) to achieve complete replacement and minimize diluting effects between the two solutions. After the solution change, another set of currents was acquired using the identical voltage clamp protocol. This process was replicated at different time intervals (1 and 30 min) following the solution change.

### Statistical analysis

Current–voltage plots were derived from the recordings. Here, mean currents ± SEM obtained at pre‐ and post‐treatment conditions, expressed as current densities normalized to the pipette's tip area (derived from the measured pipette diameter), were subject to statistical analysis, through one‐way ANOVA plus post hoc Tukey tests using GraphPad Prism software version 6 for Windows. The differences between the sham and exposed groups were tested through a *t*‐test using the same software (*p* < 0.05). Sample sizes were described as *N*_1_ (number of brains), *N*_2_ (number of slices), and *n* (number of patches). They illustrate that the experimental procedures ensured that the studied patches were derived from a significant number of brains and their resulting slices. All statistics are based on *n*.

## RESULTS

### Exposure to a 1 mT 50 Hz EMF decreases inward and transient outward current

To investigate responses of CA1 pyramidal neurons to acute exposure to 50 Hz EMFs, pyramidal neuron cell bodies in the stratum pyramidalis were studied using the loose patch clamp method. This technique records currents from a larger membrane area than in single‐channel current recordings by the conventional tight‐seal patch clamp method. It is, therefore, more amenable to experiments involving magnetic field exposure. A baseline set of currents was initially acquired. In the test group, the EMF exposure (E) was then initiated. Currents were then recorded at various time points (15, 30, 45, 60 min) after this initiation. A sham group (S) was subjected to the same experimental conditions, but with zero applied field.

**FIGURE 2:**
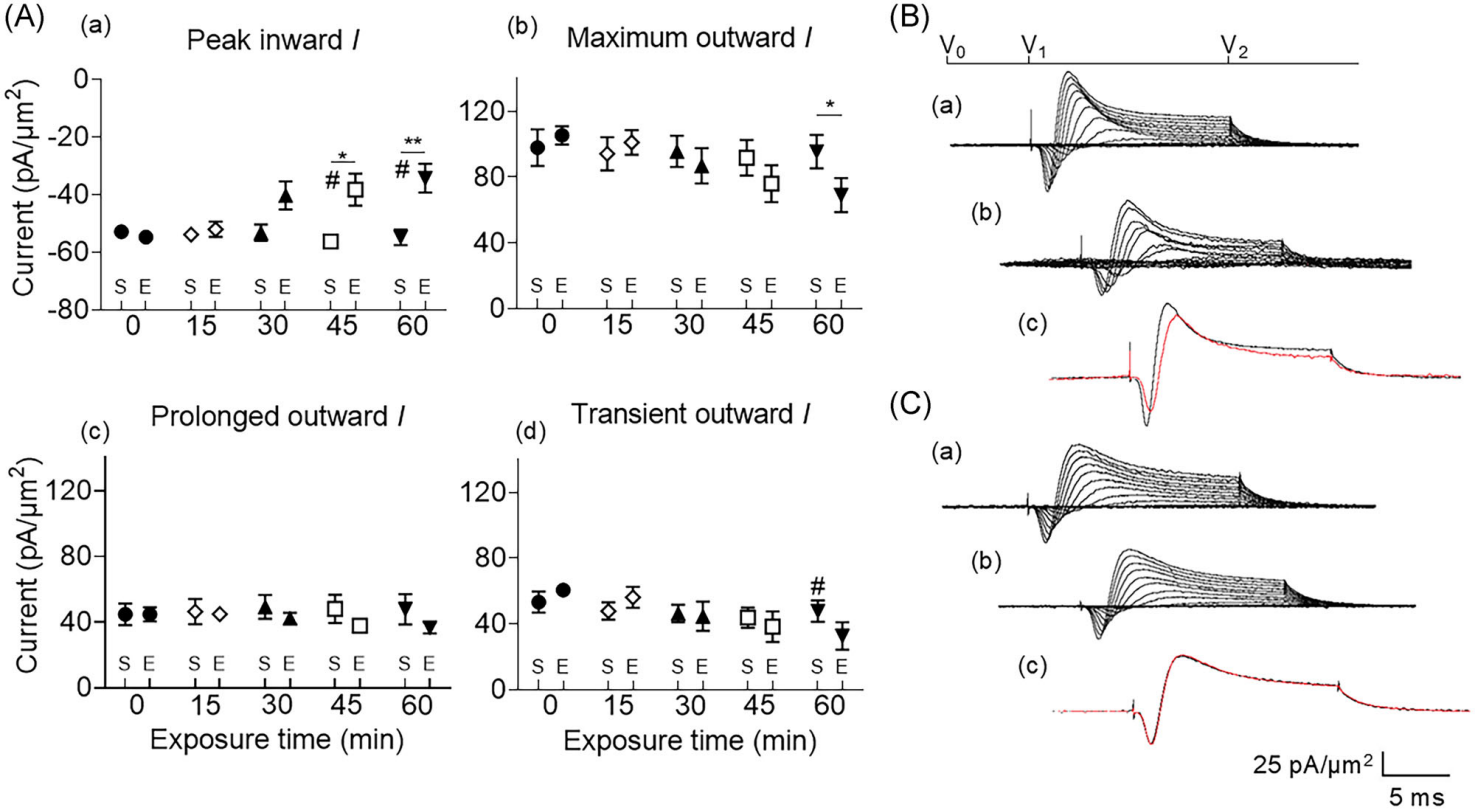
EMF exposure decreases inward and transient outward currents (*I*). Comparison between exposed and sham groups. (A) Mean (± SEM) peak currents obtained at the most depolarized voltage inspected (RMP + 120) mV in the sham (S) and exposed (E) groups are shown, expressed as current density normalized to the pipette tip diameter for baseline (black circles), 15 (empty rhombuses), 30 (black triangles), 45 (empty reversed triangles), and 60 min exposure (black rhombuses) in (a), (b), (c), and (d), respectively, for peak inward, maximum outward, prolonged outward, and transient outward currents. * represents pre‐ and post‐exposure significance, while # displays significance with respect to sham. *^,^^#^ *p* < 0.05; **p < 0.01. (B) Currents elicited by progressively depolarizing voltage steps are shown for an example patch (R_seal_: 270 kΩ, pipette diameter: 22 µm) in (a) pre‐treatment conditions and (b) following 60 min exposure to 1 mT 50 Hz electromagnetic fields (EMFs). Subpanel (c) displays single trace comparisons between control (black trace) and exposure (red trace) at the most depolarized voltage tested, (RMP + 120) mV. (C) Currents elicited by progressively depolarizing voltage steps are shown for an example patch (R_seal_: 290 kΩ, pipette diameter: 22 µm) in (a) pre‐treatment conditions and (b) following 60 min sham exposure. Subpanel (c) displays single trace comparisons between control (black trace) and sham exposure (red trace) at the most depolarized voltage tested, (RMP + 120) mV. Average R_seal_: 310 kΩ. RMP, resting membrane potential. *N*_1_ = 7, *N*_2_ = 7, *n* = 7 (for EMF), *N*_1_ = 5, *N*_2_ = 5, *n* = 5 (for sham).

The test group showed a progressive decrease in inward currents (Figure 2), detectable from 30 minutes. It reached statistical significance at 45 min, with a lower recorded value at 60 min (mean: 54.68 ± 0.77 pA/µm^2^ pre‐exposure vs. 34.27 ± 4.95 pA/µm^2^ post‐exposure, *p* = 0.0225, *n* = 7; Table 1). The transient component of the outward current was similarly significantly decreased after 60 min exposure (mean: 60.67 ± 3.57 pA/µm^2^ pre‐exposure vs. 32.59 ± 8.37 pA/µm^2^ post‐exposure, *p* = 0.0411, *n* = 7). The prolonged component of the outward current did not show significant decreases (mean: 44.69 ± 4.22 pA/µm^2^ pre‐exposure vs. 36.42 ± 3.32 pA/µm^2^ post‐exposure, *p* = 0.3319, *n* = 7).

**TABLE 1:** Summary of results of maximum current amplitude pre‐ and post‐treatment (expressed as mean ± SEM) for each experimental condition and current component tested.

Condition	Current component	Before (mean ± SEM)	After (mean ± SEM)	*n*	*p* value
**EMF 50 Hz, 1 mT (60 min)**	PI	54.68 ± 0.77	34.27 ± 4.95	7	0.0225
	MO	105.4 ± 5.56	69.01 ± 10.43	7	0.067
	PO	44.69 ± 4.22	36.42 ± 3.32	7	0.3319
	TO	60.67 ± 3.57	32.59 ± 8.37	7	0.0411
**EMF (sham)**	PI	52.08 ± 1.21	54.77 ± 2.7	5	0.7381
	MO	97.93 ± 11.07	95.48 ± 10.14	5	0.7382
	PO	44.68 ± 6.59	47.77 ± 9.17	5	0.473
	TO	53.25 ± 6.42	47.7 ± 6.45	5	0.6824
**Dantrolene 10 µM + EMF**	PI	52.56 ± 1.71	46.82 ± 5.67	5	0.6858
	MO	79.93 ± 4.45	73.09 ± 7.07	5	0.7339
	PO	32.04 ± 3.48	29.09 ± 2.94	5	0.8602
	TO	47.89 ± 6.93	44 ± 7.84	5	0.7468
**CPA 1 µM + EMF**	PI	53.51 ± 1.08	51.62 ± 3.89	6	0.9756
	MO	96.5 ± 7.12	88.47 ± 7.5	6	0.5919
	PO	45.12 ± 3.38	44.96 ± 3.11	6	> 0.9999
	TO	51.38 ± 4.15	43.5 ± 4.95	6	0.497

*Note*: *I*_max_ is expressed as current density normalized to the area of the patch (pA/µm^2^). Significance of paired differences between results obtained before and following solution change is expressed as the *p* value obtained from one way ANOVA + post hoc Tukey test for the other experimental conditions.

Abbreviations: CPA, cyclopiazonic acid; EMF, electromagnetic field; MO, maximum outward current; PI, peak inward current; PO, prolonged outward current; TO, transient outward current.

The sham group contrastingly showed no change in peak inward currents (mean: 52.08 ± 1.21 pA/µm^2^ pre‐exposure vs. 54.77 ± 2.7 pA/µm^2^ post‐exposure, *p* = 0.7381, *n* = 5). Similarly, it showed no differences in either the amplitude of the transient outward (mean: 53.25 ± 6.42 pA/µm^2^ pre‐exposure vs. 47.7 ± 6.45 pA/µm^2^ post‐exposure, *p* = 0.6824, *n* = 5) or the prolonged outward current (mean: 44.68 ± 6.59 pA/µm^2^ pre‐exposure vs. 47.77 ± 9.17 pA/µm^2^ post‐exposure, *p* = 0.473, *n* = 5).

### RyR antagonism abrogates the magnetic field effects

**FIGURE 3:**
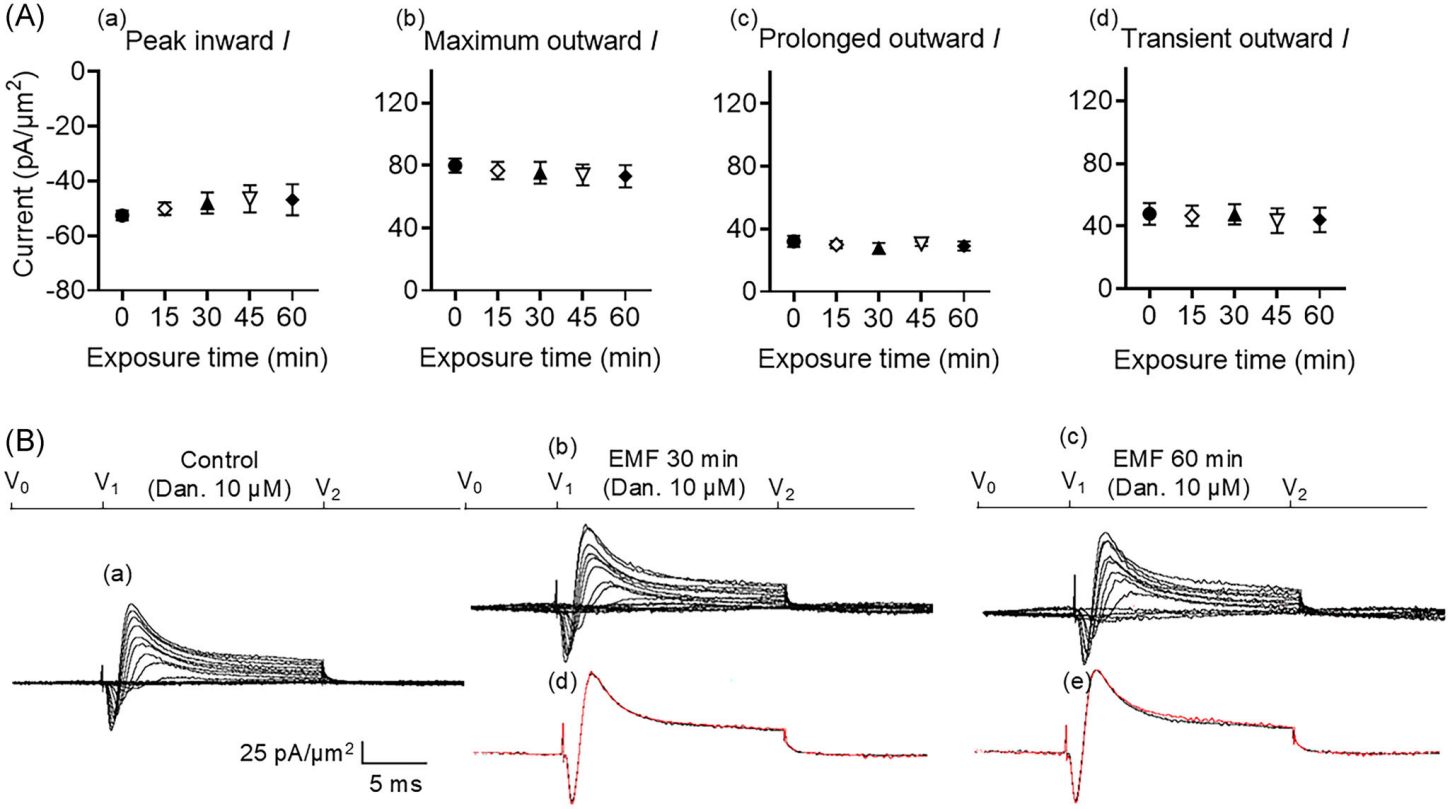
Activation properties of inward and outward currents of murine neurons in the CA1 hippocampus under loose patch clamp in response to exposure to 1 mT 50 Hz electromagnetic fields (EMFs) and previous administration of 10 µM of the RyR blocker dantrolene (Dan.). (A) Mean (± SEM) peak currents at the most depolarized voltage inspected (RMP + 120) mV, expressed as current density normalized to the pipette tip diameter, are shown for baseline (black circles), EMF 15 min (empty rhombuses), EMF 30 min (black triangles), EMF 45 min (empty reversed triangles), and EMF 60 min (black rhombuses) in (a), (b), (c), and (d), for peak inward, maximum outward, prolonged outward, and transient outward currents, respectively. (B) Currents elicited by progressively depolarizing voltage steps are shown for an example patch (R_seal_: 260 kΩ, pipette diameter: 22 µm) in (a) pre‐treatment control conditions and following exposure to a 1 mT 50 Hz EMF at (b) 30 min and (c) 60 min. Subpanels (d) and (e) display single trace comparisons between control (black trace) and treatment (red trace) at the most depolarized voltage tested, (RMP + 120) mV, respectively, after 30 and 60 min of exposure. Average R_seal_: 300 kΩ. RMP, resting membrane potential. *N*_1_ = 6, *N*_2_ = 6, *n* = 6.

In a previous report, pre‐administration of the RyR inhibitor dantrolene (10 µM) by itself left subsequently recorded inward and outward currents unaltered. But it abrogated changes in such currents following a challenge by the RyR activator caffeine. The latter actions were attributed to their sensitivity to caffeine‐induced Ca^2+^ release.^35^ Here, dantrolene (10 µM) pre‐incubation similarly abrogated the effects of the 50 Hz EMF (Figure 3). Dantrolene thus did not alter peak inward current, even at the later 60 min time point (mean: 52.56 ± 1.71 pA/µm^2^ pre‐exposure vs. 46.82 ± 5.67 pA/µm^2^ post‐exposure, *p* = 0.6858, *n* = 5). In the presence of dantrolene, EMF exposure did not alter either the transient (mean: 47.89 ± 6.93 pA/µm^2^ pre‐exposure vs. 44 ± 7.84 pA/µm^2^ post‐exposure, *p* = 0.7468), or the prolonged outward currents (mean: 32.04 ± 3.48 pA/µm^2^ pre‐exposure vs. 29.09 ± 2.94 pA/µm^2^ post‐exposure, *p* = 0.8602). These findings implicate intracellular ER calcium release in the actions of the EMF.

### SERCA block abrogates the effect of magnetic fields

**FIGURE 4:**
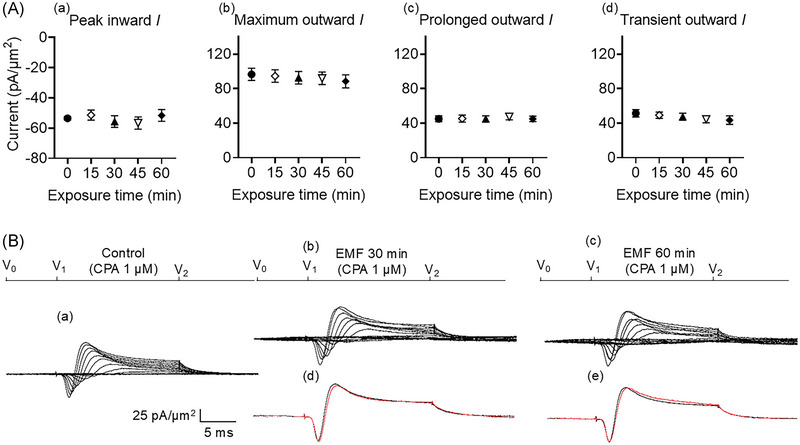
Activation properties of inward currents and outward currents of murine neurons in the CA1 hippocampus under loose patch clamp in response to exposure to 1 mT 50 Hz electromagnetic fields (EMFs) and previous administration of 1 µM of the SERCA blocker CPA. (A) Mean (± SEM) peak currents at the most depolarized voltage inspected (RMP + 120) mV, expressed as current density normalized to the pipette tip diameter, are shown for baseline (black circles), EMF 15 min (empty rhombuses), EMF 30 min (black triangles), EMF 45 min (empty reversed triangles), and EMF 60 min (black rhombuses) in (a), (b), (c), and (d), for peak inward, maximum outward, prolonged outward, and transient outward currents, respectively. (B) Currents elicited by progressively depolarizing voltage steps are shown for an example patch (R_seal_: 250 kΩ, pipette diameter: 22 µm) in (a) pre‐treatment control conditions, and following exposure to a 1 mT 50 Hz EMF at (b) 30 min and (c) 60 min. Subpanels (d) and (e) display single trace comparisons between control (black trace) and treatment (red trace) at the most depolarized voltage tested, (RMP + 120) mV, respectively, after 30 and 60 min of exposure. Average R_seal_: 290 kΩ. RMP, resting membrane potential. *N*_1_ = 6, *N*_2_ = 6, *n* = 6.

The previous experiments similarly reported that the SERCA inhibitor CPA (1 µM) pre‐administration by itself left both inward and outward currents unchanged. However, in common with dantrolene, it abrogated the effects of subsequent 0.5 mM caffeine challenges on both inward and outward currents.^35^ Here, CPA similarly abrogated the action of the EMF in decreasing inward current (mean: 53.51 ± 1.08 pA/µm^2^ pre‐exposure vs. 51.62 ± 3.89 pA/µm^2^ post‐exposure, *p* = 0.9756, *n* = 6) induced by the magnetic fields (Figure 4). The EMF now produced no significant decrease in transient outward current (mean: 51.38 ± 4.15 pA/µm^2^ pre‐exposure vs. 43.5 ± 4.95 pA/µm^2^ post‐exposure, *p* = 0.4970, *n* = 5). There were no differences in prolonged outward current (mean: 45.12 ± 3.38 pA/µm^2^ pre‐exposure vs. 44.96 ± 3.11 pA/µm^2^ post‐exposure, *p* > 0.9999, *n* = 5).

## DISCUSSION

We explored the effects of 50 Hz EMFs on CA1 pyramidal neuron membrane currents in hippocampal brain slices. Using the loose patch clamp configuration avoided perturbations of Ca^2+^ homeostasis associated with the use of Ca^2+^ buffers such as ethylene glycol‐bis (β‐aminoethyl ether)‐*N*, *N*, *N*’, *N*’‐tetra acetic acid (EGTA) to optimize seal stability in conventional patch clamping.^43^, ^44^ The effects of EMFs are known to vary with frequency, intensity,^45^, ^46^ and exposure time.^1^ We accordingly adopted a single, relatively high field intensity (1 mT). At this intensity, EMFs were previously reported to produce diverse effects on neuronal tissue physiology.^14^, ^25^, ^41^, ^47^ We also employed exposure durations previously associated with actions on [Ca^2+^]_i_.^25^

Currents were recorded at 15‐min intervals and compared with pre‐exposure results. No significant effects were observed at ≤15 min. However, from 30 min, amplitudes of both inward and transient outward currents progressively decreased, reaching a ∼ 40% decrease in inward and a ∼ 50% decrease in the transient outward current at later time points. There were no effects in the sham group studied under the same experimental conditions, but in the absence of EMF. Previous studies in rat CA1 pyramidal neurons had reported similar outward current changes^48^ but increased inward currents with EMF. However, these had employed single‐cell patch clamp containing Ca^2+^‐chelating EGTA and F^−^ in the internal pipette solution. The present studies contrastingly employed loose patch–clamped intact cells with intact Ca^2+^ homeostatic mechanisms.

Possible roles for cellular Ca^2+^ homeostasis in these effects of EMF were tested through investigating the effects of the RyR blocker dantrolene and SERCA blocker CPA. In a previous report, the RyR agonist caffeine (0.5 mM)—known to increase background [Ca^2+^]_i_, similar to the EMF described here—was shown to decrease inward and transient outward currents, findings attributed to their sensitivity to Ca^2+^ release.^35^ In contrast, dantrolene (10 µM) pre‐administration itself left such subsequently recorded inward and outward currents unaltered. However, it then abrogated EMF effects on both the inward and transient outward currents. This parallels previous reported effects of dantrolene, such as blocking the population spike amplification exerted by steady magnetic fields on hippocampal preparations in vitro.^49^ Similarly, CPA administration by itself did not affect the inward and outward currents, but it also abrogated the EMF‐induced effects on these currents. This agrees with previous reports that radio‐frequency magnetic fields increased [Ca^2+^]_i_ in HEK 293 cells, and that this effect was blocked by pre‐incubation with 10 µM of the SERCA blocker thapsigargin.^17^ The same pharmacological treatment abolished, in cultured entorhinal cortex neurons, the reduction in amplitude of the high‐K^+^–evoked Ca^2+^ elevation triggered by acute exposure to 50 Hz EMF.^14^

The present experiments thus demonstrate a modulation of hippocampal neuron membrane currents by applied EMF in a loose patch configuration that is abrogated by pharmacological manipulation of ER Ca^2+^ stores, reducing cytosolic relative to stored Ca^2+^. The latter implicates altered Ca^2+^ homeostasis in such actions. However, this is a complex multicomponent process involving multiple interacting signaling mechanisms. These include not only sarcoplasmic reticulum (SR) and surface membrane, such as RyRs, SERCAs, and Navs, but also cytosolic regulatory molecules, including calmodulin and related kinase molecules. Further biophysical experiments might complement the present single physiological Na^+^ current readouts to clarify their individual responses to an EMF. These could add molecular‐level understanding to the present physiological insights described here.

## AUTHOR CONTRIBUTIONS

K.J. and F.B. conceived the outline of the study. F.B. performed the experiments. F.B., S.A., C.L.‐H.H., and H.R.M. performed the data analysis. F.B. wrote the manuscript, and K.J., C.L.‐H.H., H.R.M., R.L., J.M., and S.R.P.S. edited and contributed to the manuscript. K.J. and J.M. acquired funding.

## COMPETING INTERESTS

The authors declare no competing interests.

## Data Availability

The data that support the findings of this study are available from the corresponding author upon request.

## References

[1] Bertagna, F. , Lewis, R. , Silva, S. R. P. , McFadden, J. , & Jeevaratnam, K. (2021). Effects of electromagnetic fields on neuronal ion channels: A systematic review. Annals of the New York Academy of Sciences, 1499, 82–103. 33945157 10.1111/nyas.14597

[2] Levin, M. (2003). Bioelectromagnetics in morphogenesis. Bioelectromagnetics, 24, 295–315. 12820288 10.1002/bem.10104

[3] Van Huizen, A. V. , Morton, J. M. , Kinsey, L. J. , Von Kannon, D. G. , Saad, M. A. , Birkholz, T. R. , Czajka, J. M. , Cyrus, J. , Barnes, F. S. , & Beane, W. S. (2019). Weak magnetic fields alter stem cell–mediated growth. Science Advances, 5, eaau7201. 30729158 10.1126/sciadv.aau7201 PMC6353618

[4] Ahmad, M. , Galland, P. , Ritz, T. , Wiltschko, R. , & Wiltschko, W. (2007). Magnetic intensity affects cryptochrome‐dependent responses in *Arabidopsis thaliana* . Planta, 225, 615–624. 16955271 10.1007/s00425-006-0383-0

[5] Pesce, M. , Patruno, A. , Speranza, L. , & Reale, M. (2013). Extremely low frequency electromagnetic field and wound healing: Implication of cytokines as biological mediators. European Cytokine Network, 24, 1–10. 23674517 10.1684/ecn.2013.0332

[6] Gualdi, G. , Costantini, E. , Reale, M. , & Amerio, P. (2021). Wound repair and extremely low frequency‐electromagnetic field: Insight from in vitro study and potential clinical application. International Journal of Molecular Sciences, 22, 5037. 34068809 10.3390/ijms22095037 PMC8126245

[7] Li, J. , Luo, Y. , & Pu, K. (2021). Electromagnetic nanomedicines for combinational cancer immunotherapy. Angewandte Chemie International Edition, 60, 12682–12705. 32671893 10.1002/anie.202008386

[8] Schuermann, D. , & Mevissen, M. (2021). Manmade electromagnetic fields and oxidative stress—Biological effects and consequences for health. International Journal of Molecular Sciences, 22, 3772. 33917298 10.3390/ijms22073772 PMC8038719

[9] Siebner, H. R. , Funke, K. , Aberra, A. S. , Antal, A. , Bestmann, S. , Chen, R. , Classen, J. , Davare, M. , Di Lazzaro, V. , Fox, P. T. , Hallett, M. , Karabanov, A. N. , Kesselheim, J. , Beck, M. M. , Koch, G. , Liebetanz, D. , Meunier, S. , Miniussi, C. , Paulus, W. , … Ugawa, Y. (2022). Transcranial magnetic stimulation of the brain: What is stimulated?–A consensus and critical position paper. Clinical Neurophysiology, 140, 59–97. 35738037 10.1016/j.clinph.2022.04.022 PMC9753778

[10] Moya Gómez, A. , Font, L. P. , Brône, B. , & Bronckaers, A. (2021). Electromagnetic field as a treatment for cerebral ischemic stroke. Frontiers in Molecular Biosciences, 8, 742596. 34557522 10.3389/fmolb.2021.742596 PMC8453690

[11] Zhang, W. , Deng, B. , Xie, F. , Zhou, H. , Guo, J.‐F. , Jiang, H. , Sim, A. , Tang, B. , & Wang, Q. (2022). Efficacy of repetitive transcranial magnetic stimulation in Parkinson's disease: A systematic review and meta‐analysis of randomised controlled trials. EClinicalMedicine, 52, 101589. 35923424 10.1016/j.eclinm.2022.101589 PMC9340539

[12] Zymantiene, J. , Juozaitiene, V. , Zelvyte, R. , Oberauskas, V. , Spancerniene, U. , Sederevicius, A. , & Aniuliene, A. (2020). Effect of electromagnetic field exposure on mouse brain morphological and histopathological profiling. Journal of Veterinary Research, 64, 319–324. 32587921 10.2478/jvetres-2020-0030 PMC7305646

[13] Leung, A. , He, C.‐Q. , Gao, Q. , Yang, Y.‐H. , Lau, B.‐M. , Wang, Q. , Liao, L.‐Y. , & Xie, Y.‐J. (2021). Extremely low frequency electromagnetic fields promote cognitive function and hippocampal neurogenesis of rats with cerebral ischemia. Neural Regeneration Research, 16, 1252–1257. 33318402 10.4103/1673-5374.301020 PMC8284293

[14] Luo, F.‐L. , Yang, N. , He, C. , Li, H.‐L. , Li, C. , Chen, F. , Xiong, J.‐X. , Hu, Z.‐A. , & Zhang, J. (2014). Exposure to extremely low frequency electromagnetic fields alters the calcium dynamics of cultured entorhinal cortex neurons. Environmental Research, 135, 236–246. 25462671 10.1016/j.envres.2014.09.023

[15] Sun, Z.‐C. , Ge, J. , Guo, B. , Guo, J. , Hao, M. , Wu, Y. , Lin, Y. , La, T. , Yao, P. , Mei, Y. , Feng, Y. , & Xue, L. (2016). Extremely low frequency electromagnetic fields facilitate vesicle endocytosis by increasing presynaptic calcium channel expression at a central synapse. Scientific Reports, 6, 1–11. 26887777 10.1038/srep21774 PMC4757866

[16] Lisi, A. , Ledda, M. , Rosola, E. , Pozzi, D. , Emilia, E. D. , Giuliani, L. , Foletti, A. , Modesti, A. , Morris, S. J. , & Grimaldi, S. (2006). Extremely low frequency electromagnetic field exposure promotes differentiation of pituitary corticotrope‐derived AtT20 D16V cells. Bioelectromagnetics, 27, 641–651. 16838272 10.1002/bem.20255

[17] Bertagna, F. , Lewis, R. , Silva, S. R. P. , McFadden, J. , & Jeevaratnam, K. (2022). Thapsigargin blocks electromagnetic field‐elicited intracellular Ca2+ increase in HEK 293 cells. Physiological Reports, 10, e15189. 35510320 10.14814/phy2.15189 PMC9069166

[18] Tenforde, T. S. , & Kaune, W. (1987). Interaction of extremely low frequency electric and magnetic fields with humans. Health Physics, 53, 585–606. 3679823 10.1097/00004032-198712000-00002

[19] Fedorowski, A. , & Steciwko, A. (1998). Biological effects of non‐ionizing electromagnetic radiation. Medycyna Pracy, 49, 93–105. 9587915

[20] World Health Organization . (2007). Electromagnetic fields and public health .

[21] Komaki, A. , Khalili, A. , Salehi, I. , Shahidi, S. , & Sarihi, A. (2014). Effects of exposure to an extremely low frequency electromagnetic field on hippocampal long‐term potentiation in rat. Brain Research, 1564, 1–8. 24727530 10.1016/j.brainres.2014.03.041

[22] Balassa, T. , Varró, P. , Elek, S. , Drozdovszky, O. , Szemerszky, R. , Világi, I. , & Bárdos, G. (2013). Changes in synaptic efficacy in rat brain slices following extremely low‐frequency magnetic field exposure at embryonic and early postnatal age. International Journal of Developmental Neuroscience, 31, 724–730. 24012627 10.1016/j.ijdevneu.2013.08.004

[23] Cuccurazzu, B. , Leone, L. , Podda, M. V. , Piacentini, R. , Riccardi, E. , Ripoli, C. , Azzena, G. B. , & Grassi, C. (2010). Exposure to extremely low‐frequency (50 Hz) electromagnetic fields enhances adult hippocampal neurogenesis in C57BL/6 mice. Experimental Neurology, 226, 173–182. 20816824 10.1016/j.expneurol.2010.08.022

[24] Panagopoulos, D. J. , Karabarbounis, A. , & Margaritis, L. H. (2002). Mechanism for action of electromagnetic fields on cells. Biochemical and Biophysical Research Communications, 298, 95–102. 12379225 10.1016/s0006-291x(02)02393-8

[25] Morabito, C. , Guarnieri, S. , Fan& Ograve, G. , & Mariggi& Ograve, M. A. (2010). Effects of acute and chronic low frequency electromagnetic field exposure on PC12 cells during neuronal differentiation. Cellular Physiology and Biochemistry, 26, 947–958. 21220925 10.1159/000324003

[26] He, Y.‐L. , Liu, D.‐D. , Fang, Y.‐J. , Zhan, X.‐Q. , Yao, J.‐J. , & Mei, Y.‐A. (2013). Exposure to extremely low‐frequency electromagnetic fields modulates Na+ currents in rat cerebellar granule cells through increase of AA/PGE2 and EP receptor‐mediated cAMP/PKA pathway. PLoS ONE, 8, e54376. 23349866 10.1371/journal.pone.0054376 PMC3551899

[27] Marchionni, I. , Paffi, A. , Pellegrino, M. , Liberti, M. , Apollonio, F. , Abeti, R. , Fontana, F. , D'inzeo, G. , & Mazzanti, M. (2006). Comparison between low‐level 50 Hz and 900 MHz electromagnetic stimulation on single channel ionic currents and on firing frequency in dorsal root ganglion isolated neurons. Biochimica Et Biophysica Acta (BBA)‐Biomembranes, 1758, 597–605. 16713990 10.1016/j.bbamem.2006.03.014

[28] Yin, C. , Luo, X. , Duan, Y. , Duan, W. , Zhang, H. , He, Y. , Sun, G. , & Sun, X. (2016). Neuroprotective effects of lotus seedpod procyanidins on extremely low frequency electromagnetic field‐induced neurotoxicity in primary cultured hippocampal neurons. Biomedicine & Pharmacotherapy, 82, 628–639. 27470406 10.1016/j.biopha.2016.05.032

[29] Manikonda, P. K. , Rajendra, P. , Devendranath, D. , Gunasekaran, B. , Channakeshava , Aradhya, R. S. S. , Sashidhar, R. B. , & Subramanyam, C. (2007). Influence of extremely low frequency magnetic fields on Ca2+ signaling and NMDA receptor functions in rat hippocampus. Neuroscience Letters, 413, 145–149. 17196332 10.1016/j.neulet.2006.11.048

[30] Pozzan, T. , Rizzuto, R. , Volpe, P. , & Meldolesi, J. (1994). Molecular and cellular physiology of intracellular calcium stores. Physiological Reviews, 74, 595–636. 8036248 10.1152/physrev.1994.74.3.595

[31] Nicotera, P. , & Orrenius, S. (1998). The role of calcium in apoptosis. Cell Calcium, 23, 173–180. 9601613 10.1016/s0143-4160(98)90116-6

[32] de Groot, M. W. , van Kleef, R. G. , de Groot, A. , & Westerink, R. H. (2016). In vitro developmental neurotoxicity following chronic exposure to 50 Hz extremely low‐frequency electromagnetic fields in primary rat cortical cultures. Toxicological Sciences, 149, 433–440. 26572663 10.1093/toxsci/kfv242

[33] de Groot, M. W. , Kock, M. D. , & Westerink, R. H. (2014). Assessment of the neurotoxic potential of exposure to 50 Hz extremely low frequency electromagnetic fields (ELF‐EMF) in naive and chemically stressed PC12 cells. Neurotoxicology, 44, 358–364. 25111744 10.1016/j.neuro.2014.07.009

[34] Bertagna, F. , Ahmad, S. , Lewis, R. , Silva, S. R. P. , Mcfadden, J. , Huang, C. L.‐H. , Matthews, H. R. , & Jeevaratnam, K. (2024). Loose patch clamp membrane current measurements in cornus ammonis 1 neurons in murine hippocampal slices. Annals of the New York Academy of Sciences, 1535, 62–75. 38602714 10.1111/nyas.15123

[35] Bertagna, F. , Ahmad, S. , Lewis, R. , Silva, S. R. P. , Mcfadden, J. , Huang, C. L.‐H. , Matthews, H. R. , & Jeevaratnam, K. (2024). Loose‐patch clamp analysis applied to voltage‐gated ionic currents following pharmacological ryanodine receptor modulation in murine hippocampal cornu ammonis‐1 pyramidal neurons. Frontiers in Physiology, 15, 1359560. 38720787 10.3389/fphys.2024.1359560 PMC11076846

[36] Matthews, H. R. , Tan, S. R. X. , Shoesmith, J. A. , Ahmad, S. , Valli, H. , Jeevaratnam, K. , & Huang, C. L.‐H. (2019). Sodium current inhibition following stimulation of exchange protein directly activated by cyclic‐3′, 5′‐adenosine monophosphate (Epac) in murine skeletal muscle. Scientific Reports, 9, 1927. 30760734 10.1038/s41598-018-36386-0 PMC6374420

[37] King, J. H. , Wickramarachchi, C. , Kua, K. , Du, Y. , Jeevaratnam, K. , Matthews, H. R. , Grace, A. A. , Huang, C. L.‐H. , & Fraser, J. A. (2013). Loss of Nav1. 5 expression and function in murine atria containing the RyR2‐P2328S gain‐of‐function mutation. Cardiovascular Research, 99, 751–759. 23723061 10.1093/cvr/cvt141

[38] Salvage, S. C. , Habib, Z. F. , Matthews, H. R. , Jackson, A. P. , & Huang, C. L.‐H. (2021). Ca2+‐dependent modulation of voltage‐gated myocyte sodium channels. Biochemical Society Transactions, 49, 1941–1961. 34643236 10.1042/BST20200604 PMC8589445

[39] Salvage, S. C. , Dulhunty, A. F. , Jeevaratnam, K. , Jackson, A. P. , & Huang, C. L.‐H. (2023). Feedback contributions to excitation–contraction coupling in native functioning striated muscle. Philosophical Transactions of the Royal Society B, 378, 20220162. 10.1098/rstb.2022.0162 PMC10150225 37122213

[40] Sarbjit‐Singh, S. S. , Matthews, H. R. , & Huang, C. L.‐H. (2020). Ryanodine receptor modulation by caffeine challenge modifies Na+ current properties in intact murine skeletal muscle fibres. Scientific Reports, 10, 2199. 32042141 10.1038/s41598-020-59196-9 PMC7010675

[41] Liu, S. X. , Matthews, H. R. , & Huang, C. L.‐H. (2021). Sarcoplasmic reticular Ca2+‐ATPase inhibition paradoxically upregulates murine skeletal muscle Nav1. 4 function. Scientific Reports, 11, 2846. 33531589 10.1038/s41598-021-82493-w PMC7854688

[42] Ting , J. T. , Daigle, T. L. , Chen, Q. , & Feng, G. (2014). Acute brain slice methods for adult and aging animals: Application of targeted patch clamp analysis and optogenetics. Methods in Molecular Biology, 1183, 221–242. 25023312 10.1007/978-1-4939-1096-0_14 PMC4219416

[43] Leech, C. A. , & Holz, G. G., IV (1994). Application of patch clamp methods to the study of calcium currents and calcium channels. Methods in Cell Biology, 40, 135–151. 8201974 10.1016/s0091-679x(08)61113-9 PMC3509330

[44] Perkins, K. L. (2006). Cell‐attached voltage‐clamp and current‐clamp recording and stimulation techniques in brain slices. Journal of Neuroscience Methods, 154, 1–18. 16554092 10.1016/j.jneumeth.2006.02.010 PMC2373773

[45] Simkó, M. , & Mattsson, M. O. (2004). Extremely low frequency electromagnetic fields as effectors of cellular responses in vitro: Possible immune cell activation. Journal of Cellular Biochemistry, 93, 83–92. 15352165 10.1002/jcb.20198

[46] Girgert, R. , Schimming, H. , Körner, W. , Gründker, C. , & Hanf, V. (2005). Induction of tamoxifen resistance in breast cancer cells by ELF electromagnetic fields. Biochemical and Biophysical Research Communications, 336, 1144–1149. 16168388 10.1016/j.bbrc.2005.08.243

[47] Zhou, J. , Ming, L.‐G. , Ge, B.‐F. , Wang, J.‐Q. , Zhu, R.‐Q. , Wei, Z. , Ma, H.‐P. , Xian, C. J. , & Chen, K.‐M (2011). Effects of 50 Hz sinusoidal electromagnetic fields of different intensities on proliferation, differentiation and mineralization potentials of rat osteoblasts. Bone, 49, 753–761. 21726678 10.1016/j.bone.2011.06.026

[48] Zheng, Y. , Xia, P. , Dong, L. , Tian, L. , & Xiong, C. (2021). Effects of modulation on sodium and potassium channel currents by extremely low frequency electromagnetic fields stimulation on hippocampal CA1 pyramidal cells. Electromagnetic Biology and Medicine, 40, 274–285. 33594919 10.1080/15368378.2021.1885433

[49] Wieraszko, A. (2000). Dantrolene modulates the influence of steady magnetic fields on hippocampal evoked potentials in vitro. Bioelectromagnetics, 21, 175–182. 10723017 10.1002/(sici)1521-186x(200004)21:3<175::aid-bem4>3.0.co;2-3

